# Loneliness as a symptom of major depression in youth: a cohort study

**DOI:** 10.21203/rs.3.rs-11322343/v1

**Published:** 2026-10-22

**Authors:** João Villanova Amaral, Gabriele dos Santos Jobim, Christian Kieling, João Pedro Gonçalves Pacheco, Lucas Toshio Ito, Rafaella Ormond, Marcos Leite Santoro, Síntia Iole Belangero, Ary Gadelha, Euripedes Constantino Miguel, Rodrigo Affonseca Bressan, Pedro Mario Pan, Luis Augusto Rohde, Giovanni Abrahão Salum, Mauricio Scopel Hoffmann

## Abstract

This study evaluated loneliness as an associate symptom and severity indicator of major depressive disorder (MDD) in adolescents and young adults. We used data from the Brazilian High-Risk Cohort Study when participants were aged 14–23 (n = 1,905) and 18–28 years (n = 2,052). We examined loneliness centrality in a depressive symptom network and evaluated its role as a severity indicator by comparing clinical groups defined by clinician-diagnosed MDD and the presence of loneliness. Four groups were established: MDD with loneliness, MDD without loneliness, loneliness without MDD, and neither MDD nor loneliness. Cross-sectional and longitudinal associations with life-threatening outcomes (suicide attempt, psychiatric admission, death), poor life chances (criminal convictions, substance use, NEET), and well-being were assessed using regression models adjusted for sociodemographic factors and social isolation. Loneliness was part of the depressive construct and the network's most central symptom. Individuals with MDD and loneliness showed higher concurrent odds of life-threatening outcomes (OR: 3.963, 95% CI: 2.571, 6.177) and lower well-being (B: -2.100, 95% CI: -3.096, -1.104), than those depressed without loneliness. Prospectively, this group showed poorer well-being (B: -1.477, 95% CI: -2.420, -0.534). These findings support loneliness as an associate symptom and severity indicator of MDD. Assessing loneliness may improve clinical characterization and help identify young people at greater risk of persistent impairment.

